# EUPopLink Country report - Georgia

**DOI:** 10.12688/openreseurope.23973.1

**Published:** 2026-05-29

**Authors:** Sandro Tabatadze, Bacho Khuroshvili

**Affiliations:** 1 Assistant Professor, Political Science, Ivane Javakhishvili Tbilisi State University, Tbilisi, Tbilisi, Georgia; 2 PhDc, Institute of Political Science, University of Wrocław, Wrocław, Lower Silesian Voivodeship, Poland

**Keywords:** Populism, Euroscepticism, Georgia, EU, EU candidate state, Georgian Dream

## Abstract

This country report examines the relationship between populism and Euroscepticism in Georgia, a case in which support for European integration has long constituted one of the most stable elements of public and political consensus. It traces how this consensus has become increasingly politicised under conditions of democratic erosion, geopolitical insecurity, and the Russia–Ukraine war. The report argues that Euroscepticism in Georgia has moved from the political margins to the centre of state power, through its growing articulation by populist political actors. While the Conservative Movement/Alt-Info articulates hard Euroscepticism through anti-liberal, nativist and openly pro-Russian narratives, the ruling Georgian Dream has developed a softer but increasingly assertive form of conditional Euroscepticism. Instead of opposing EU membership directly, Georgian Dream repackages European integration in terms of sovereignty, peace, tradition, dignity, and resistance to external coercion. Moreover, the ruling party’s populist rhetoric portrays itself as the guardian of “the people” against all domestic forces that oppose its policies, as well as liberal civil society, the embassies of other states in Georgia, and the EU elites themselves. In this context, Euroscepticism becomes not merely a foreign-policy position, but a populist instrument of regime legitimation and authoritarian consolidation.

## 1. Introduction

The present country report was prepared as part of the EUPopLink COST Action ^1^ following the EUPopLink Theoretical Framework and guidelines. ^2^

Since Georgia declared its independence from the Soviet Union in 1991, Westernization and European integration have consistently been central to the political and public agenda. ^3^ However, this issue was overshadowed by civil wars from 1991 to 1993 and a severe socio-economic crisis. It was not until 1995, when Eduard Shevardnadze, the former Minister of Foreign Affairs of the USSR and a Georgian diplomat, came to power, that the EU began to be seen as a partner for Georgia. ^4^ Nonetheless, high levels of corruption and internal political instability, along with a pragmatic balancing act with Russia, made European integration seem unrealistic to both the political elite and the public. ^5^

The situation shifted dramatically after the Rose Revolution in 2003, a bloodless revolt that brought pro-Western young reformers to power in Georgia. The declared objective was to integrate the nation into Euro-Atlantic frameworks. ^6^ This strategic initiative fostered a closer relationship with the EU, and public opinion surveys revealed a resounding endorsement of this trajectory, with more than seventy-five percent of the population in agreement. ^7^ However, Saakashvili’s post-revolutionary government adopted a fierce policymaking approach and sometimes set overly ambitious expectations for the population. ^8^ Consequently, in the later years of his presidency, many citizens began to feel disillusioned about European integration.

In 2012, Georgia experienced a democratic transition of power for the first and only time through elections. Despite accusations of having pro-Russian tendencies, the new government made European integration a key foreign policy priority, which was supported by the majority of the opposition and an overwhelming proportion of the population. In every parliamentary and presidential election, candidates from the ruling party and the main opposition promised closer ties with the EU. ^9^ This was evident in the signing of the Association Agreement in 2014, the establishment of the Deep and Comprehensive Free Trade Agreement (DCFTA), the introduction of 90-day visa-free travel to the Schengen Area, and the granting of EU candidate status. ^10^ However, this trend has vividly shifted in recent years, signaling a sudden and unsettling change in foreign policy from the ruling party.

## 2. Populist political actors

In Georgia, populist political actors have traditionally had a limited influence. They were primarily composed of small political groups, many of which have not registered as official political parties. ^11^ Recently, however, new populist parties have emerged, and some established parties have adopted populist ideologies. ^12^ Below is a brief profile of each of these actors.

• **The Labor Party** is a left-wing populist party that has been active since 1995 and has consistently maintained its place in opposition. At various points, it has successfully gained entry into parliament, while at other times, it has operated exclusively as a non-parliamentary opposition. ^13^ The party’s populist nature is evident in how its leaders portray themselves as the primary champions of the interests of the oppressed and impoverished, standing against a political elite composed of wealthy individuals, oligarchs, and bankers. ^14^
• Small political unions like **Georgian March, Georgian Idea, and Georgian National Unity** were established between 2015 and 2016. These groups are known for their far-right and anti-immigration views. ^15^ They have consistently struggled to achieve electoral success and are considered politically marginal. Their ideology is grounded in exclusive populism, promoting nativist ideas that prioritize the interests of ethnically Georgian Orthodox Christians, whom they believe deserve special attention due to their relatively small numbers. ^16^
• **The Alliance of Patriots of Georgia** is a far-right populist party established in 2012 with the objective of thwarting the resurgence of liberal forces in power. ^17^ The party presents itself as a defender of Georgian Orthodox citizens and traditional values, actively opposing the political elite of both the current and past governments. While they achieved third place in the 2016 parliamentary elections with 5% of the vote, their involvement in public politics has diminished since that time. ^18^
• **The Conservative Movement/Alt-Info** was founded in 2021 as a media outlet and transitioned into a political party in 2024. This organization embodies right-wing populism, portraying itself as the primary defender of the interests of the Georgian people against foreigners, Western liberals, migrants from North Africa and Asia, as well as global non-governmental and international organizations. ^19^ The party espouses anti-Western rhetoric and seeks to promote amicable relations between Georgia and Russia. ^17^ Following government interference in registration, they contested the 2024 parliamentary elections under the Patriotic Alliance of Georgia. According to the Central Election Commission results, they garnered 2.53% of the vote.
• **The Georgian Dream** (GD) party was founded in 2012 as a catch-all political movement and initially acted as an observer for the Party of European Socialists. ^20^ However, over the years, the ruling party has increasingly adopted characteristics of right-wing populism, presenting itself as the primary defender of national and sovereign interests against the global elite. In the wake of the Russia-Ukraine war in 2022, GD has advanced the conspiracy theory that decisions made by the U.S. and the EU are heavily influenced by a faction they call the “Global War Party.” GD contends that this so-called Global War Party, akin to a deep state, consists of self-proclaimed liberals who oppose national interests and aim to undermine the Georgian government. They argue that this undermining is intended to coerce Georgia into conflict with Russia and ultimately facilitate greater control of the Georgian populace by the global elite. ^21^ Initially, a faction of the GD known as “People’s Power” expressed these strong positions. This faction formally separated from the ruling party in 2022, aiming to “reveal the truth to the people”. ^22^ However, in the 2024 parliamentary elections, its members decided to rejoin the GD list to participate in the elections.

## 3. Positions on ‘Europe’ and the Populism-Euroscepticism nexus

Only three of the populist political actors previously discussed are currently active: the Labor Party, the Conservative Movement/Alt Info, and the Georgian Dream. Thus, this section will address these parties exclusively. It is worth noting that the **Labor Party** is pro-European and cannot be considered Eurosceptic. However, it sometimes criticizes the European political elite for reluctance to address issues with the Georgian government. ^23^ Therefore, the discussion will first focus on the extra parliamentary opposition party, followed by the ruling party.

**The Conservative Movement/Alt-Info** initially established a media outlet before evolving into a political party in 2024, primarily operating through online platforms. The party holds a *hard Eurosceptic* stance, asserting that Georgia is unlikely to join the EU and that further debate on this issue is unnecessary. They oppose the current policies of the EU, as well as the foundational principles upon which it was established. Leaders of Alt-Info compare the EU to the Soviet Union, viewing it as a centralized entity that undermines the sovereignty of nation-states while imposing a liberal agenda on its member countries. ^24^

The party exhibits *cultural and political Euroscepticism*, opposing what they label the “Brussels dictatorship” and the EU’s positions on issues such as abortion, gender equality, diversity, and migration. According to Alt-Info leaders, Georgian and European values are fundamentally incompatible. They assert that a significant portion of the population seeks European integration primarily for economic benefits, believing they are being misled. ^25^ Some party members even use the term “Europeanists” in a sarcastic manner, equating it with a sense of powerlessness.

Moreover, Alt-Info leaders frequently reference Brexit to reinforce their views, envisioning an ideal Europe comprised of independent nation-states. The party has firmly opposed the EU’s COVID-19 policies, including vaccinations and the implementation of green cards. Their support for Georgia’s rapprochement with Russia is evident, as they sometimes visit the Kremlin. ^26^ Since the onset of the Russian-Ukrainian war, they have claimed that the Zelensky government was misled by the West, insisting that Ukraine will ultimately face defeat. The party advocates for Georgia to adopt a neutral stance in this conflict to avoid a scenario reminiscent of the Georgian Rose Revolution or the Maidan protests. ^27^

Leaders of Alt-Info have openly accused the Joe Biden administration and EU officials of fostering the “Ukrainianization” of Georgia. They contend that this revolutionary scenario could lead to conflict with Russia. From their perspective, Donald Trump’s reelection and his relationships with Russia and other world leaders suggest that the influence of globalists and liberals is dwindling—a development they view positively.

In summary, within its populist framework, the EU is perceived as part of an oppressive elite, while ethnically Georgian Orthodox individuals are seen as an oppressed group. Alt-Info argues that only a government prioritizing national interests, such as their own, can effectively safeguard these interests.

**Georgian Dream** (GD) is a *soft Eurosceptic* party that supports Georgia’s potential accession to the EU, albeit under specific circumstances. This position allows it to be characterized as conditionally Eurosceptic. In the 2024 parliamentary elections, the ruling party adopted the motto: “Towards Europe only with peace, only with dignity, only with prosperity”. ^28^

The party also engages in Eurosceptic rhetoric, asserting that Georgia has no political lessons to learn from the corrupt bureaucrats in Brussels and advocating for restraint in political statements aimed solely at gaining favor. Members of the GD frequently label most MEPs as corrupt, lacking principles, and lacking substantial influence. ^27^

Furthermore, GD exhibits elements of *cultural Euroscepticism*, particularly concerning issues such as gender equality and LGBTQ rights. Their vision of an ideal Europe is reminiscent of the perspectives held by Viktor Orbán and Robert Fico. They emphasize minimal influence from Brussels and prioritize common values identified by the chairman of GD—namely, respect for God, homeland, and family. ^28^

Until 2022, before the outbreak of the Russo-Ukrainian war, the GD exhibited a fragmented and predominantly reactionary form of Euroscepticism. While the party generally supported European integration, it often remained silent when faced with specific criticisms akin to the behavior of a sulky child. Its stance on issues like Brexit and Covid-19 was typically in alignment with the EU. However, following Russia’s invasion of Ukraine, the ruling party announced that it would not participate in the sanctions imposed by the EU against Russia and would refrain from openly supporting Ukraine. ^21^ This decision was rooted in a fear of Russia and concerns expressed by some EU officials regarding the potential for opening a second front in the conflict, which ultimately became a significant cornerstone of GD’s Eurosceptic position.

Since that period, the party has been criticizing the practice and future of the EU, as well as accusing it of double standards. According to the GD conspiracy, representatives of the Global War Party (a formation similar to the deep state) are in the ruling elites of both the EU and some of its states, and it was they who planned to start a revolution in Georgia and then drag it into war with Russia. ^21^ Therefore, the ruling team declared itself oppressed and said that only it could defend the interests of the Georgian people against the oppressive elite, including some influential representatives of the EU, which is a good example of the combination of populism and Euroscepticism.

Since 2022, the Euroscepticism demonstrated by the GD, particularly following the involvement of representatives from certain EU countries and its own members, has taken on a more pronounced tone. Rather than reflecting the behavior of an innocent child, it now resembles that of a smug teenager. ^21^ This style of Euroscepticism remains reactionary but is assertive enough to dismiss numerous concerns with a sweeping motion.

Furthermore, after Donald Trump secured his victory in the United States, the GD claimed that a strategic partnership with the US would soon be restored due to “ideological convergence”. ^28^ It was subsequently followed by a letter from Prime Minister Irakli Kobakhidze to President Trump, in which he conveyed wishes for Trump to prevail over the so-called “deep state,” as the Georgian government did it with the “Global War Party” elite. However, the current US administration has yet to respond.

## 4. Responses to Euroscepticism and consequences

The pro-European opposition, which vigorously counters the Eurosceptic narrative, characterizes both the Conservative Movement/Alt-Info and Georgian Dream not only as openly Eurosceptic but also as pro-Russian forces, effectively creating the “cordon sanitare” around them. ^29^ Consequently, European integration has become the focal point of public-political discussions.

However, the current state of modern Georgia is alarming, with a notable erosion in democratic norms and a consolidation of authoritarianism ^30^ Several members of the pro-European opposition are imprisoned, while others face severe financial constraints or are at risk of incarceration. As a result, despite their “adversarial” response to the anti-European sentiment, the influence of the pro-European elite remains limited.

As for the concrete consequences of populist political actors’ Euroscepticism, both foreign and domestic political dimensions are important here. In this regard, it is worth noting:

• Termination of European integration by Georgia’s ruling party;
• The emergence of new partners (Hungary and Slovakia in Europe, Iran and China in Asia);
• Sanctions by some EU countries on the Georgian government and individuals close to them;
• Suspension of ongoing educational, agricultural, and cultural projects by the EU and its member states;
• Public confrontation between the embassies of the EU and several of its member states and the GD;
• The Georgian ruling party’s refusal to allow some EU citizens with anti-government statements to cross the border;
• Incitement of conspiratorial populist Eurosceptic positions among the ruling party’s supporters;
• Strengthening democratic erosion involves imposing restrictions on assembly, expression, and other civil liberties, as well as actively persecuting political opponents. It includes new legislation on foreign agents, media censorship, limitations on wearing masks during protests and abolishing the concept of gender, among other measures.

These results align with GD’s populist narrative, which is linked to their Euroscepticism.

## Conclusion

The Georgian case is particularly noteworthy because Euroscepticism and populism have long remained outside the political mainstream, historically confined to marginal non-parliamentary actors. Since 2012, however, the EU dimension has become progressively politicized as right-wing populist narratives - rooted in sovereignty and cultural protectionism - found an ideal target in the EU’s value-based conditionality. This alignment enabled these actors, including eventually the ruling party, to reframe Europe not simply as a strategic destination but as a political battleground. Non-populist actors, while consistently defending integration, have also contributed to the heightened salience of the EU issue: their criticism of democratic erosion provides populists with further ammunition to depict them as agents of “external pressure.” The Russia-Ukraine war intensified these dynamics, allowing Euroscepticism to move from the margins into the core of state policy.

Therefore, several key avenues for future research arise from this case. First, the Russia-Ukrainian war can be seen as a crisis that has accelerated the growth of populist Euroscepticism, sparking new ideas within this context across nearly all European countries. Thus, the urgency of examining the effects of the Russia-Ukrainian war on this phenomenon cannot be overstated.

Second, analyzing Georgia as a case of rapid transformation and contrasting it with other Eastern European nations, such as Romania—where pro-European forces have demonstrated significant democratic resilience—can provide important insights.

Third, the populist and Eurosceptic rhetoric of both the Conservative Movement/Alt-Info and the Georgian Dream, seemingly influenced by policy transfers from Hungary, presents a rich area for further exploration. This exploration, combined with comparative public policy concepts like policy learning, policy transfer, and lesson drawing, could yield valuable insights.

Lastly, conducting a systematic comparative study between the Western Balkan states and the Eastern European trio—old and new candidates for EU membership—would contribute to a deeper understanding of the political dynamics within EU candidate member states.

## Ethics and consent

Ethical approval and consent were not required.

## Data Availability

No data are associated with this article.
