# Supplementary material for: EUPopLink Country report - Georgia
Source: Open Res Eur. 2026 Jul 23;6:185. Originally published 2026 May 29. [Version 2] doi: 10.12688/openreseurope.23973.2 (PMC13555050; doi:10.12688/openreseurope.23973.2)
Supplement: Peer review history

# Reviewer report: Reviewer response for version 1

**DOI:** 10.21956/openreseurope.25953.r75398

**Published:** 2026-07-15

**Authors:** Pernille Rieker

**Affiliations:** 1 ARENA – Centre for European Studies at the University of Oslo, Oslo, Norway

The article provides a rich and timely analysis of how populist actors in Georgia have progressively mainstreamed Euroscepticism, transforming it from a marginal stance into a central instrument of regime legitimation and authoritarian consolidation. The empirical narrative is detailed and well-structured, especially in tracing the evolution from pro‑European consensus to conditional and hard Eurosceptic positions within the ruling party and newer far‑right actors. The distinction between hard Euroscepticism (Alt‑Info) and conditional, sovereignty‑focused Euroscepticism (Georgian Dream) is analytically valuable, and the linkage to wider dynamics such as democratic erosion and the Russia–Ukraine war is convincing. At the same time, some of the more ambitious claims—particularly regarding the depth of policy reorientation and the precise mechanisms through ˆwhich populist rhetoric reshapes foreign‑policy choices—would benefit from clearer conceptual operationalization and more explicit engagement with the broader comparative literature on populism, external governance, and EU candidate states.

 Overall, the report is a strong and useful contribution that opens important avenues for future comparative research, including the role of crises, policy transfer from Hungary and other illiberal governments, and the differentiated trajectories of Euroscepticism across Eastern Europe and the Western Balkans.

Is the case presented with sufficient detail to be useful for teaching or other practitioners?

Yes

Is the work clearly and accurately presented and does it cite the current literature?

Yes

If applicable, is the statistical analysis and its interpretation appropriate?

Yes

Are all the source data underlying the results available to ensure full reproducibility?

Yes

Are the conclusions drawn adequately supported by the results?

Yes

Is the background of the case’s history and progression described in sufficient detail?

Yes

Reviewer Expertise:

Political science, security studies, eu studies

I confirm that I have read this submission and believe that I have an appropriate level of expertise to confirm that it is of an acceptable scientific standard.

# Reviewer report: Reviewer response for version 1

**DOI:** 10.21956/openreseurope.25953.r75858

**Published:** 2026-07-14

**Authors:** Eftichia Teperoglou

**Affiliations:** 1 Aristotle University of Thessaloniki, Thessaloniki, Greece

This country report provides a comprehensive and insightful analysis of the evolving relationship between populism and Euroscepticism in Georgia. As one of the traditionally most pro-European countries among the EU's Eastern partners, Georgia has experienced a gradual politicization of European integration in recent years. By linking domestic democratic backsliding with shifts in foreign policy rhetoric, the report demonstrates how European integration has evolved from a broadly shared national objective into a highly contested political issue.

 It convincingly shows how Euroscepticism has moved from the political margins to the center of political power, with the ruling Georgian Dream increasingly framing European integration through narratives of sovereignty, national dignity, traditional values, and resistance to external influence. The distinction between the hard Euroscepticism of the Conservative Movement/Alt-Info and the softer, conditional Euroscepticism promoted by the ruling party is particularly well developed and represents one of the report's key contributions. The report also demonstrates how the Russia–Ukraine war accelerated these developments, creating the political context in which populist narratives and Eurosceptic rhetoric became increasingly prominent.

 Overall, by combining historical context with theoretical perspectives on populism and Euroscepticism, the report makes a valuable contribution to our understanding of contemporary Georgian politics and offers useful insights for comparative research on EU candidate countries.

Is the case presented with sufficient detail to be useful for teaching or other practitioners?

Yes

Is the work clearly and accurately presented and does it cite the current literature?

Yes

If applicable, is the statistical analysis and its interpretation appropriate?

Not applicable

Are all the source data underlying the results available to ensure full reproducibility?

Yes

Are the conclusions drawn adequately supported by the results?

Yes

Is the background of the case’s history and progression described in sufficient detail?

Yes

Reviewer Expertise:

EU politics, Euroscepticism, populism, electoral behaviour, European Parliament elections, party system change, public opinion, survey methodology

I confirm that I have read this submission and believe that I have an appropriate level of expertise to confirm that it is of an acceptable scientific standard.

# Reviewer report: Reviewer response for version 1

**DOI:** 10.21956/openreseurope.25953.r75401

**Published:** 2026-07-06

**Authors:** Alessio Scopelliti

**Affiliations:** 1 University of Milan, Milan, Italy

The introduction provides an excellent historical overview of Georgia from independence to recent developments, effectively contextualizing the trajectories leading up to 2014 when populism and the EU matter entered the political discussion in the country.

 The second section offers a useful description of populist parties that emerged in the mid-2010s. However, for readers unfamiliarity with these parties and Georgian politics, it would be beneficial to include a figure showcasing their electoral performances from their founding to the present. This would allow readers to better grasp the parties’ relevance and trajectory over time.

 The third section addresses the core contribution of this report, by stating the populism-Euoscepticism nexus within Georgian party politics. Three out of five populist actors are described in this section. The rationale is to explore only those parties that are still active nowadays (I insist that a plot, in the previous section, showcasing parties’ electoral performance might strength this decision. Otherwise, this is a lost opportunity to explore as many actors as possible, maybe also to explore the reasons for their disappearance). Moving on to the three parties addressed in this section, I have the following comments:

- Labor Party is only briefly addressed in the analysis. While the authors recognize it to have a few Eurosceptic stances, the party is predominantly characterized as pro-European. It would be worthwhile to provide more information about the party’s – albeit minimal – skepticism toward the EU. Specifically, what aspects of EU integration did the Labor Party question, even while endorsing the broader integration project? This effort would significantly enhance the report’s contribution to discussions about parties holding multiple nuanced stances cross-cutting positions on the EU simultaneously expressing both pro- and anti-EU positions rather than adopting monolithic stances on this matter.
- The analysis of the Conservative Movement/Alt-Info is clear and highly specific. The authors explicitly identify the type of Euroscepticism (cultural and political), connecting it to populism (although it would be more beneficial to be more explicit by citing exclusionary populism) and articulate the grounds for skepticism in a clear and compelling manner. It is interesting, on the one hand, that the party envisages the EU as the Soviet Union, thus, recalling its own historical trauma. On the other hand, it does endorse Russian stance in the invasion of Ukraine, due to similarities of the government’s regime trajectory towards authoritarianism is moving to. The implications of such developments are clearly addressed in the last sections of the report.
- Regarding Georgian Dream, it would be useful to provide more explicit information about the nature of its soft Euroscepticism. The authors refer to the Populism-Euroscepticism nexus based on valence politics, specifically focusing on criticism of EU elites accused of being corrupt. More explicitly, the authors state that this matter (EU elites’ corruption) is “a good example of the combination of populism and Euroscepticism”. However, such a link does not really address the core of the Populism-Euroscepticism nexus but rather explains how the anti-elitist core feature of populism is expressed – in this case, by identifying EU elites as the enemy. There is no real explanation of how the EU is criticized substantively, but only how it is used as a “means” to articulate anti-elitism. It is not clearly articulated how this anti-elite stance translates into Euroscepticism in terms of polity or policy dimensions. I would suggest explaining this nexus more explicitly: what specific aspects of EU integration does Georgian Dream oppose? The authors already briefly mention the cultural dimension: “issues such as gender equality and LGBTQ rights”. I think the authors should dedicate more emphasis to this aspect – the cultural dimension – as it directly connects skepticism toward EU core values of equality, diversity, and inclusion with the core characteristic of exclusionary populism. This would clarify that Georgian Dream’s Euroscepticism is not merely instrumental anti-elitism (using EU elites as scapegoats) but reflects substantive opposition to EU cultural liberalism. By elaborating on the cultural dimension of Euroscepticism, the authors would demonstrate that Georgian Dream’s opposition to the EU is both strategic (anti-elite rhetoric) and ideological (rejection of liberal cultural values).

The fourth section successfully lists the consequences of populist Eurosceptic developments in Georgia, particularly the country’s drift toward authoritarianism. I have no comments on this section. Finally, the conclusion clearly identifies avenues for further research.

Is the case presented with sufficient detail to be useful for teaching or other practitioners?

Yes

Is the work clearly and accurately presented and does it cite the current literature?

Yes

If applicable, is the statistical analysis and its interpretation appropriate?

Not applicable

Are all the source data underlying the results available to ensure full reproducibility?

No source data required

Are the conclusions drawn adequately supported by the results?

Yes

Is the background of the case’s history and progression described in sufficient detail?

Yes

Reviewer Expertise:

Party Politics, Populism, Euroscepticism, Far-Right Mainstreaming

I confirm that I have read this submission and believe that I have an appropriate level of expertise to confirm that it is of an acceptable scientific standard, however I have significant reservations, as outlined above.

# Response

**Published:** 2026-07-14

**Authors:** Sandro Tabatadze

**Affiliations:** Political Science, Ivane Javakhishvili Tbilisi State University, Tbilisi, Tbilisi, Georgia

Thank you for your positive assessment and constructive comments. The updated version of the report includes three key changes:  

 Firstly, we have clarified why the Georgian Labor Party is not discussed in the chapter on the populism-Euroscepticism nexus.

 Secondly, a table of election results has been added to illustrate the performance of populist parties in the most recent elections.

 Thirdly, we have enhanced the explanation of the soft Euroscepticism associated with Georgian Dream, clarifying it.
